# Supplementary material for: Periodic splay Fréedericksz transitions in a ferroelectric nematic
Source: Nat Commun. 2025 Feb 7;16:1444. doi: 10.1038/s41467-025-55827-9 (PMC11806116; doi:10.1038/s41467-025-55827-9)
Supplement: Supplementary file 1 — Supplementary Information [file 41467_2025_55827_MOESM1_ESM.pdf]

Supplementary Information

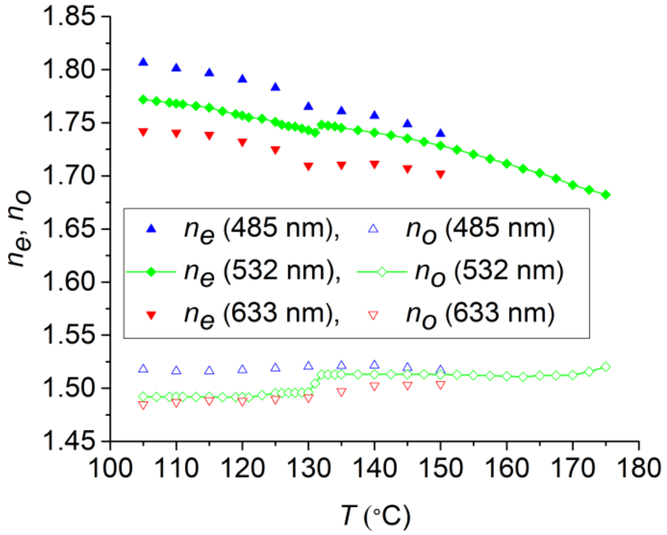

Supplementary Fig.1| Temperature dependencies of the refractive indices of RM734.

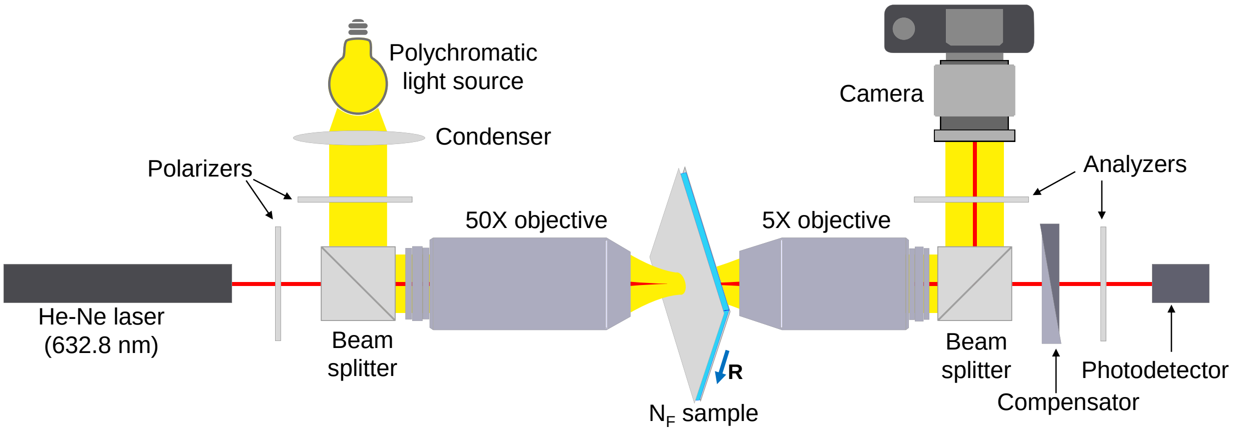

Supplementary Fig.2| Schematic diagram of the experimental setup to characterize the dynamics of electro-optical response. R is the rubbing direction of N_F cell. Incident laser light

makes an angle of 15° to the normal of the cell. The cell tilt is achieved by placing a cell on a metal prism holder.

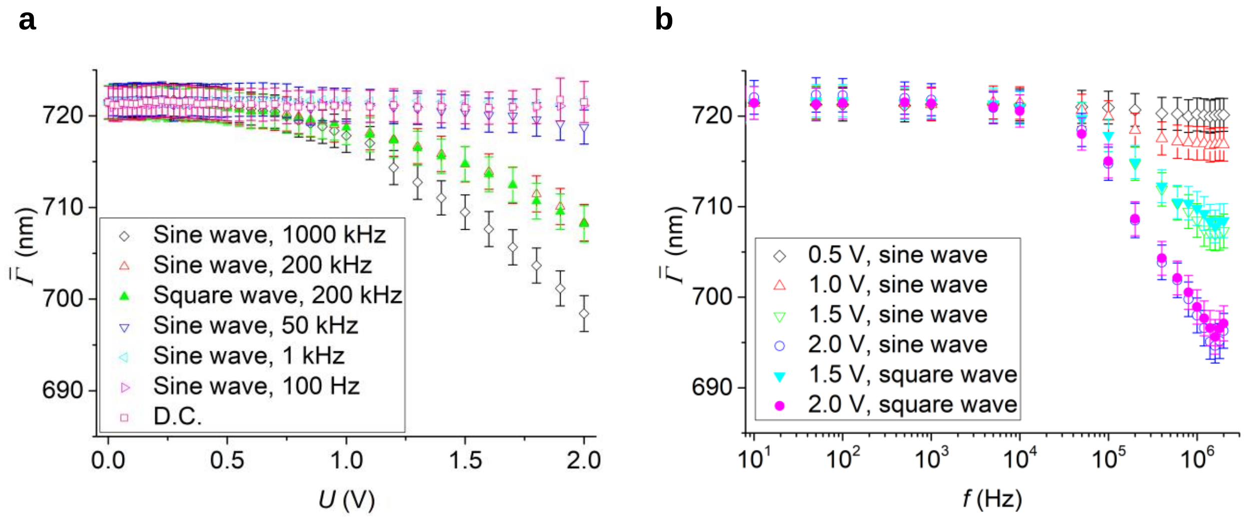

Supplementary Fig.3| Retardance change of a planar N_F cell confined between PI2555 substrates. **a,** Time-averaged retardance $\bar{\Gamma}$ vs applied voltage at different frequencies of the square and sinusoidal fields. **b,** $\bar{\Gamma}$ vs field frequency for fixed values of the rms voltage amplitude. $d = (2.9 \pm 0.1)$ µm, 125 °C. Error bars correspond to standard deviation of $\bar{\Gamma}$.

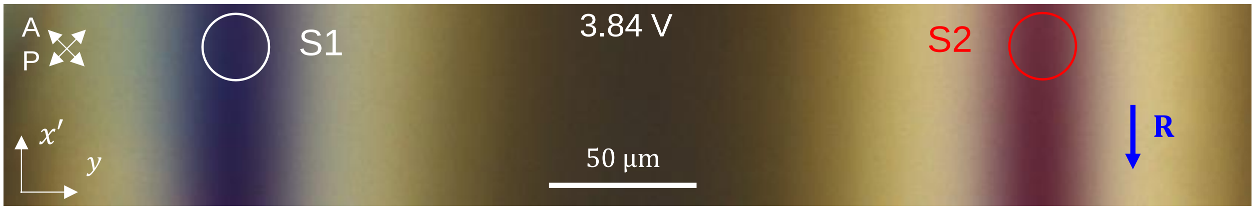

Supplementary Fig.4| Polarizing microscopy texture of splay-twist stripes in an N_F cell tilted by 45° around the *y*-axis; note different interference colors of the two edge regions with splay deformations, indicating opposite directions of the polarization tilt in S1 and S2, one towards the positive end of the z-axis, another to the negative end of the z -axis. Cell thickness $d = (2.5 \pm 0.1)$ µm, ac field, 200 kHz, square wave, 125 °C. The cell tilt is achieved by placing the cell on a metal prism holder.

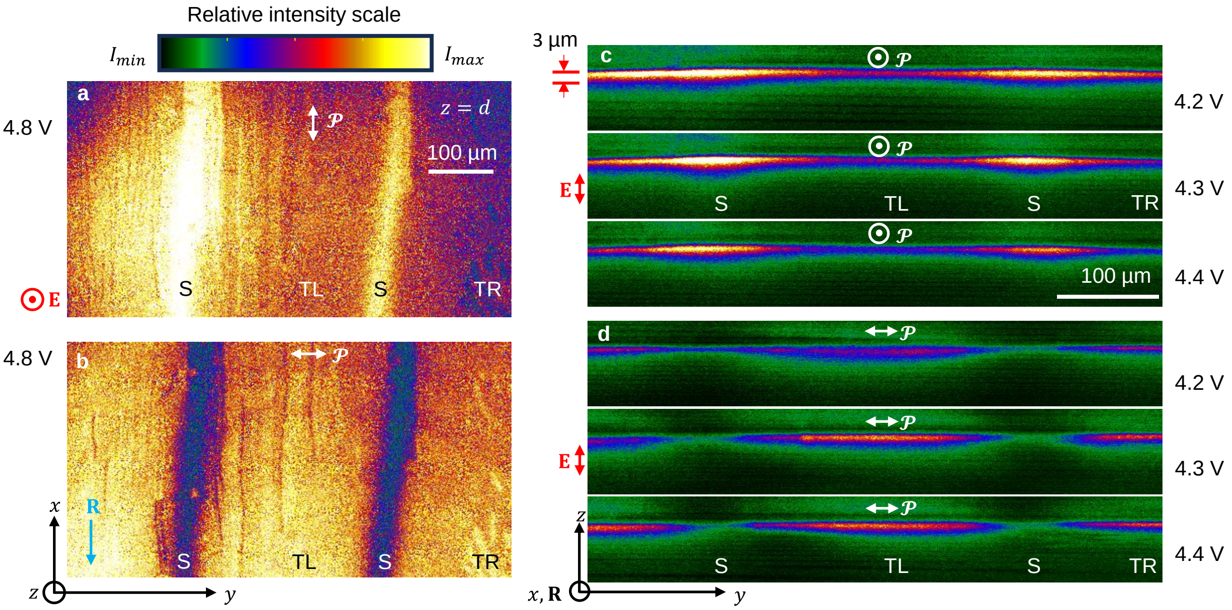

Supplementary Fig.5| Fluorescence confocal polarizing microscopy textures of the splay-twist Frederiks transition. **a,b,** Horizontal xy scans near the top plate $z = d$ for the probing light polarization $\boldsymbol{\mathcal{P}}$ parallel and perpendicular to the rubbing direction **R**, respectively. S-regions show the maximum intensity of fluorescent light when $\boldsymbol{\mathcal{P}} \parallel \mathbf{R}$ and the minimum intensity when $\boldsymbol{\mathcal{P}} \perp \mathbf{R}$. **c,d,** Vertical yz scans for $\boldsymbol{\mathcal{P}} \parallel \mathbf{R}$ and $\boldsymbol{\mathcal{P}} \perp \mathbf{R}$, respectively. The TL and TR regions show enhancement of the fluorescent signal when $\boldsymbol{\mathcal{P}} \perp \mathbf{R}$ and the voltage increases, which implies progressive deviation of the polarization vector **P** from the rubbing direction **R**. The data support the model of splay-twist in Figure 7b,c. The probing light enters the sample at the $z = d$ plane; the fluorescent signal is detected in the reflection mode. Cell thickness $d = (2.7 \pm 0.1)$ µm in (**a,b**) and (2.9 ± 0.1) µm in (**c,d**); ac field, 200 kHz, square wave, 125 °C.

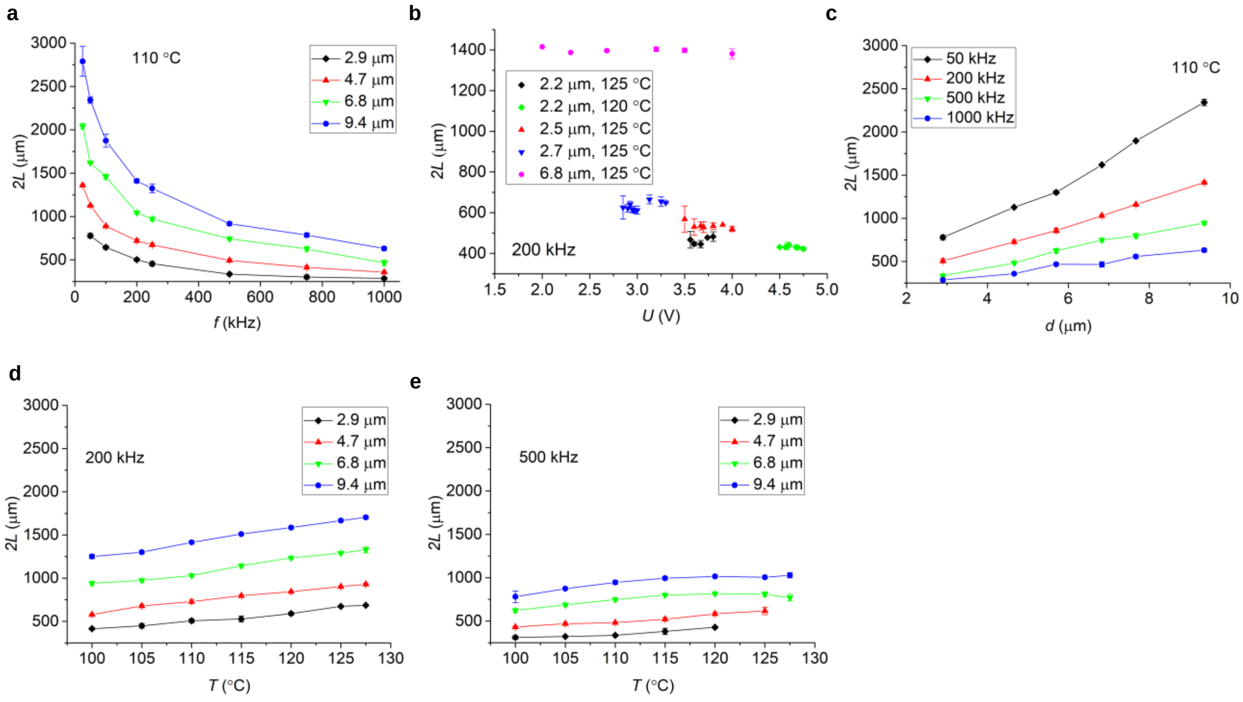

Supplementary Fig.6| Period $2L$ of the splay-twist stripes vs a, Frequency for different cell thicknesses. **b**, Amplitude of the voltage for different cell thicknesses. **c**, Cell thickness for different frequencies. **d,e,** Temperature, for the field frequencies 200 kHz and 500 kHz, respectively. Square wave ac field. Error bars represent the standard deviation of $2L$ measured within the area 2.8 mm × 1.7 mm.

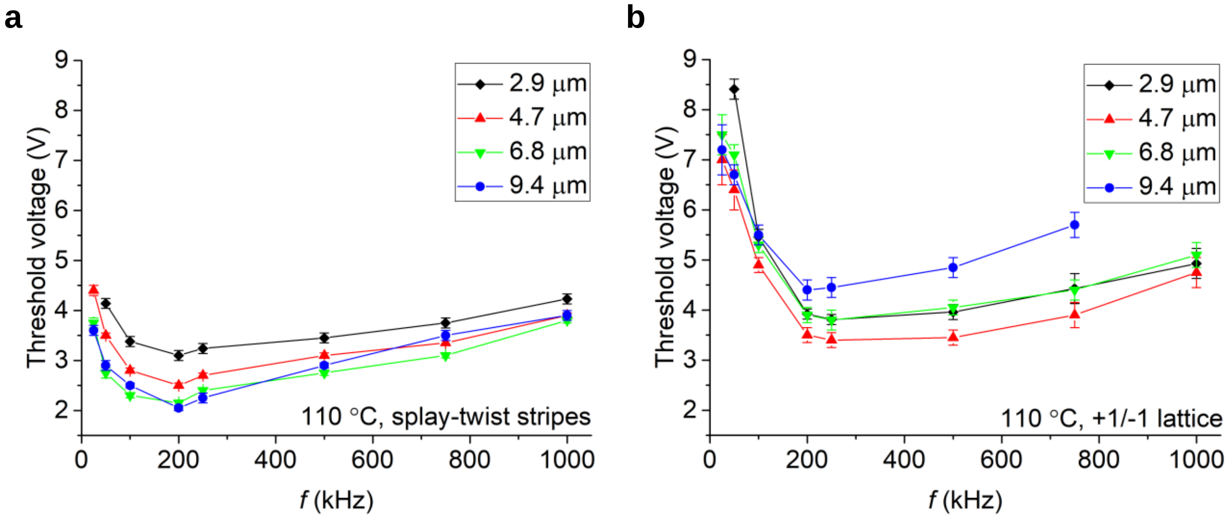

Supplementary Fig.7| Frequency dependencies of the threshold voltages for different cell thicknesses. a, U_{ST} for splay-twist stripes. **b,** U_{SB} for square lattices of +1/-1 defects. Square wave ac field; temperature 110 °C. Error bars represent the range of applied voltages within which the homogeneous oscillations around the planar orientation transition into the stationary splay-twist stripes in (**a**) and the splay-twist stripes transition into the stationary splay-bend +1/-1 lattice in (**b**).

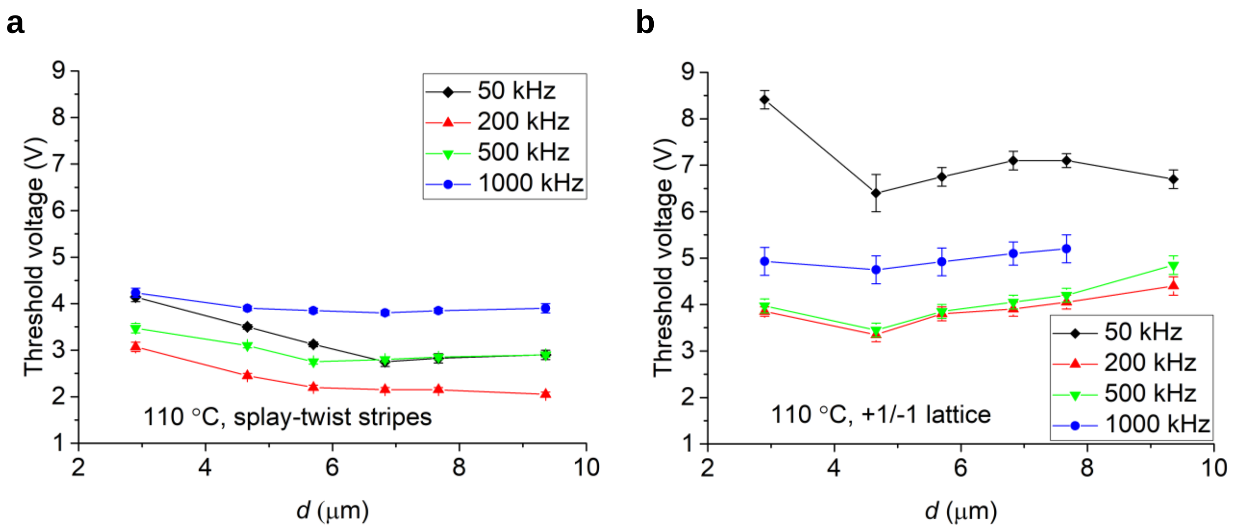

Supplementary Fig.8| Thickness dependencies of the threshold voltages for different frequencies. a, U_{ST} for splay-twist stripes. **b,** U_{SB} for square lattices of +1/-1 defects. Square wave ac field; temperature 110 °C. Error bars represent the range of applied voltages within which the homogeneous oscillations transition into the stationary splay-twist stripes in (**a**) and the splay-twist stripes transition into the stationary splay-bend +1/-1 lattice in (**b**).

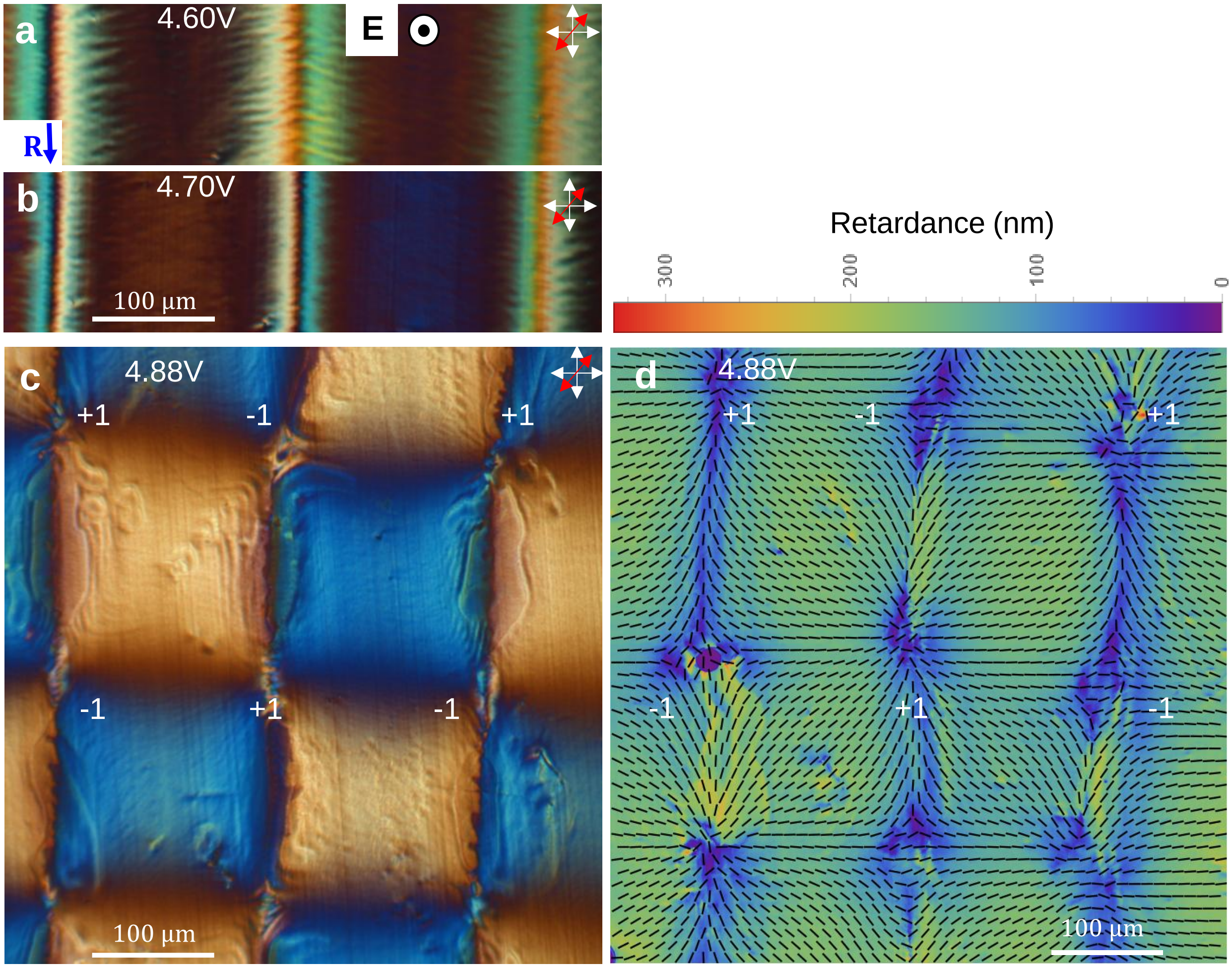

Supplementary Fig.9| Transformation from splay-twist stripes into square lattice of +1/-1 defects. a,b,c, Voltage increase causes the transformation (square wave, 200 kHz); observations with crossed polarizers and a 550 nm optical compensator (slow axis is shown by a red arrow). **d**, PolScope Microimager texture of the in-plane splay and bend; wavelength 655 nm. $d = (2.2 \pm 0.1)$ µm; 120℃.

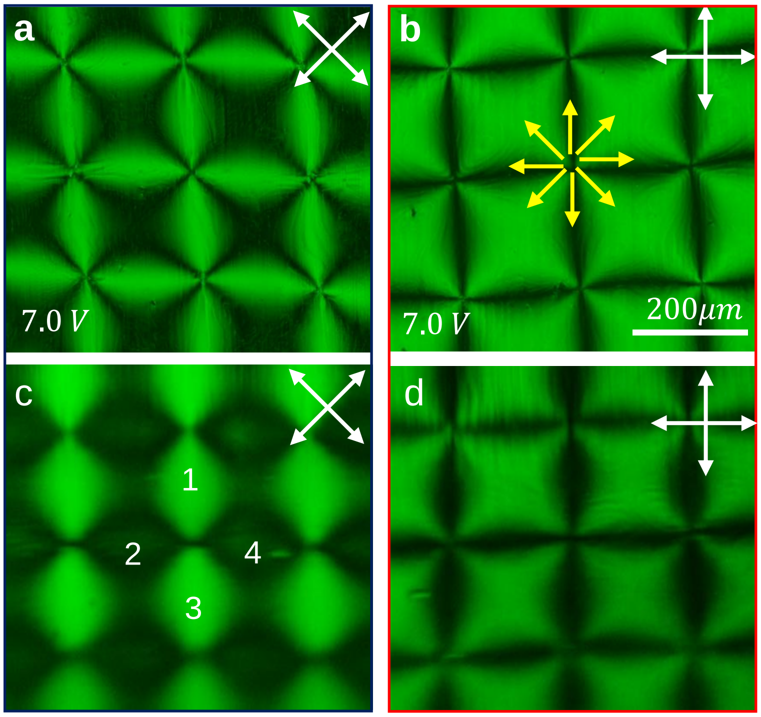

Supplementary Fig.10| Polarizing microscopy textures of +1/-1 splay-bend square lattice in an N_F cell. a,b, Monochromatic 532 nm light observations for two configuration of crossed polarizers. **c,d**, The cell is tilted around the y-axis by 45°; note the same intensity at locations 1 and 3 as well as 2 and 4 around the +1 defect in part (c); the feature is compatible with Fig.7d,e in which the top and bottom plates are the source and sink of polarization (or the other way around) and the polarization profile in the vertical planes resembles a letter C rather than S. Square wave ac field, 200 kHz, $d = (3.0 \pm 0.1)$ μm, 105 ℃.

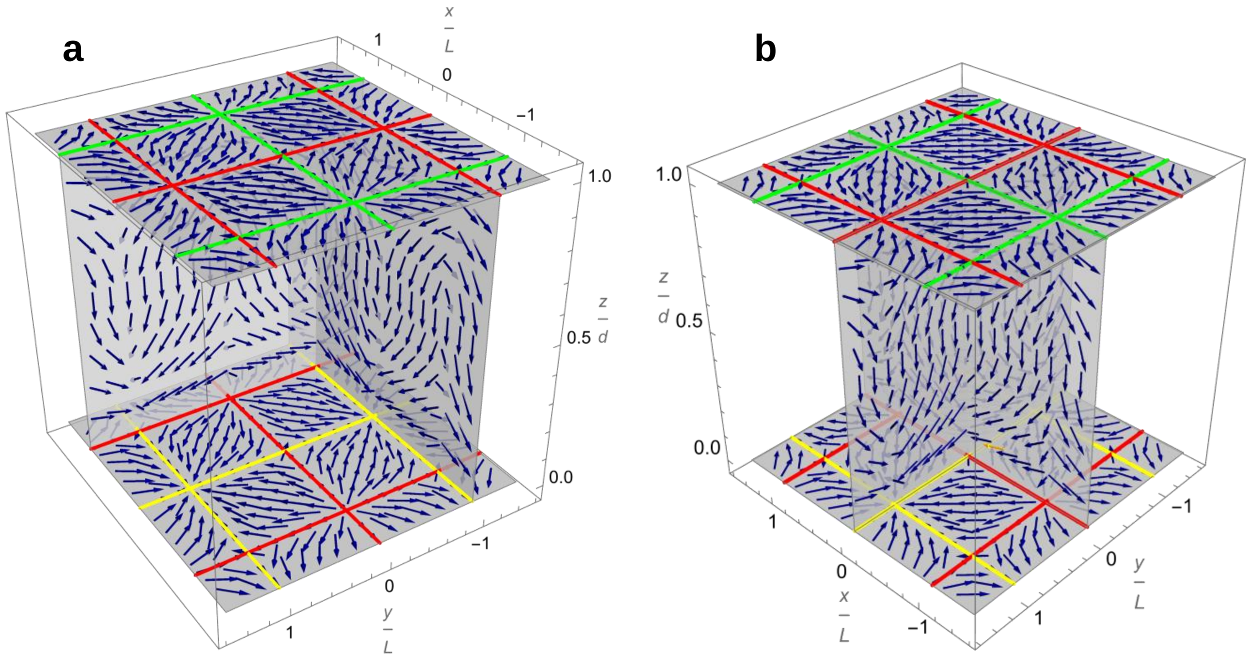

Supplementary Fig.11| Scheme of the splay-bend +1/-1 lattice in which the polarization patterns are the same at the top and bottom plates. a,b, Different vertical cross-sections of the same structure. The polarization field in the vertical planes resembles a letter S rather than C and does not realign by π, as the polarization in Fig.7d,e does. This scheme produces line regions (around red lines) in which the splay cancellation is effective; yellow and green lines mark the regions with positive and negative bound charge, respectively. Note that the separation of the positive and negative bound charges is $\sim L$, much larger than $\sim d$ in the scheme of Fig.7d,e. The polarization field is modeled as $\mathbf{P} = (P_x,\ P_y, P_z\ ) = P\left(\cos\Phi \cos\frac{\pi z}{d}, \sin\Phi \cos\frac{\pi z}{d},\ \sin\frac{\pi z}{d}\right)$, where $\Phi = \sum_{i=1,j=1}^{i=p,j=q}\left[(-1)^{i+j}\arctan\left(\frac{y+jL}{x+iL}\right)\right]$ is the superposition of the individual defect fields, p and q are the numbers of defects in rows and columns. For comparison, in Fig.7d,e, $\mathbf{P} = P\left(\cos\Phi \cos\frac{\pi z}{d}, -\sin\Phi \cos\frac{\pi z}{d}, -\sin\frac{\pi z}{d}\right)$.

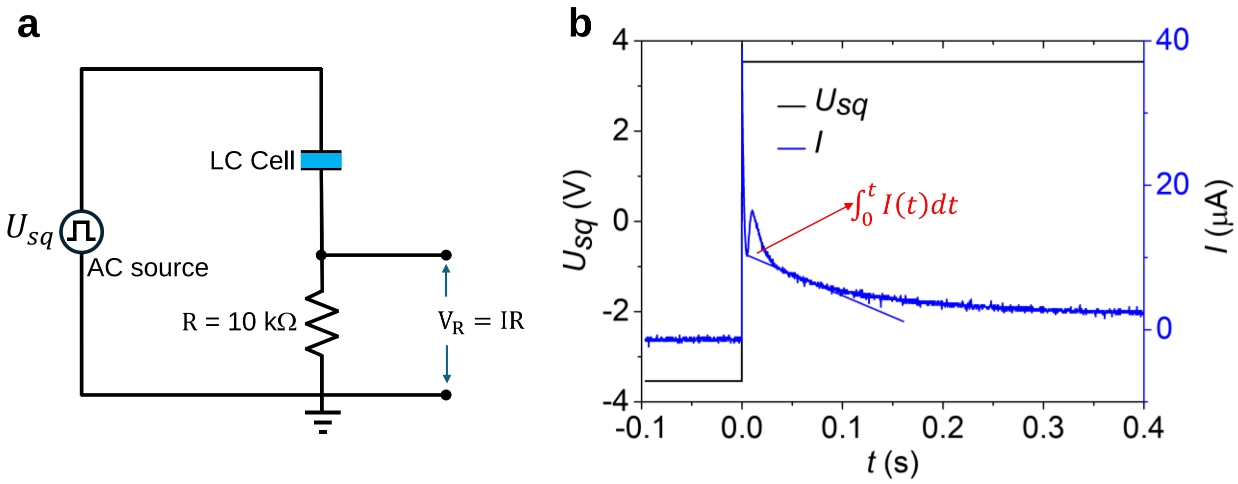

Supplementary Fig.12| Estimate of the ion concentration by voltage reversal. a, Electric circuit; 10 kΩ resistor is used to measure the current flowing in the circuit. **b,** Reversal of the applied square pulse U_{sq} and the corresponding current I as a function of time. The mobile ions produce the current bump of an area $\int_0^t I(t)dt$ after the voltage reversal. Ion concentration is calculated as $n = \frac{1}{ed\Sigma}\int_0^t I(t)\,dt = 2.4 \times 10^{20}\ \mathrm{m}^{-3}$. Electrode area Σ =1 cm^2, $d = (20.0 \pm 0.1)$ μm, 135 °C.
