# Supplementary material for: Periodic splay Fréedericksz transitions in a ferroelectric nematic
Source: Nat Commun. 2025 Feb 7;16:1444. doi: 10.1038/s41467-025-55827-9 (PMC11806116; doi:10.1038/s41467-025-55827-9)
Supplement: Supplementary file 2 — Description of Additional Supplementary Files [file 41467_2025_55827_MOESM2_ESM.pdf]

Inventory of Supplementary Information

File Name: SupplementaryInformation.pdf

Description: Supplementary information. The file includes all the supplementary figures.

File Name: Supplementary Movie 1.mp4

Description: Transition from homogeneous oscillations to splay-twist stripes. Polarizing microscopy texture of a transition from a uniformly oscillating state to the splay-twist stripes as the voltage increases from 2.5 V to 3.5 V. RM734 cell of a thickness $d = (3.2 \pm 0.1)$ µm. Sinusoidal wave, $f = 200$ kHz; 125 °C. Recording speed 10 frames per second.

File Name: Supplementary Movie 2.avi

Description: Stationary splay-twist stripe deformations. Polarizing microscopy texture of a stationary splay-twist stripe pattern in the RM734 cell of a thickness $d = (3.2 \pm 0.1)$ µm. Sinusoidal wave, $f = 200$ kHz, fixed voltage 3.0 V; 125 °C. Recording speed 10 frames per second.

File Name: Supplementary Movie 3.mp4

Description: Transition from splay-twist stripes to square lattice of splay-bend. Polarizing microscopy texture of a transition from a splay-twist stripes to splay-bend square lattice of defects as the voltage increases from 3.5 V to 6.5 V. RM734 cell of a thickness $d = (3.0 \pm 0.1)$ µm. Sinusoidal wave, $f = 200$ kHz; 115 °C. Recording speed 10 frames per second; playing speed is increased by a factor of 4.

File Name: Supplementary Movie 4.avi

Description: Electrohydrodynamic flows in a square array of +1/-1 defects. Polarizing microscopy texture of a square array of +1/-1 defects in a d = (2.2 $\pm$ 0.1) µm RM734 cell. Square wave voltage 5.31 V, $f =$ 200 kHz; 105 °C. Recording speed, 10 frames per second. Electrohydrodynamic flows are visualized by variations of the transmitted light intensity.

File Name: Supplementary Movie 5.avi

Description: Transformation from a square array of +1/-1 defects into homeotropic state and hydrodynamic flows at a high applied voltage. Polarizing microscopy texture of a $d = (3.1 \pm 0.1)$ µm cell. Sinusoidal wave, $f = 200$ kHz; 120 ℃. Applied voltage is increased from 6.5 V to 9.0 V. Recording speed, 10 frames per second. The high voltage transforms the square array of +1 and -1 defects in the N_F phase into a homeotropic state of the N phase and hydrodynamic flows.

File Name: Supplementary Movie 6.avi

Description: Transformation from a square array of +1/-1 defects into electrohydrodynamic flows at a high applied voltage. Polarizing microscopy texture of a $d = (6.8 \pm 0.1)$ µm cell. Sinusoidal wave, $f = 200$ kHz; 120 ℃. Applied voltage is increased from 6.5 V to 9.0 V. Recording speed, 10 frames per second. The high applied voltage produces strong electrohydrodynamic flows and transforms the square array of +1 and -1 defects in the N_F phase into a homeotropic N state because of the electric heating.
